# Genome Sequence of the Edible Green Alga Ulva prolifera, Originating from the Yoshinogawa River in Japan

**DOI:** 10.1128/mra.00430-22

**Published:** 2022-08-29

**Authors:** Keita Tamura, Hidemasa Bono

**Affiliations:** a Laboratory of Genome Informatics, Graduate School of Integrated Sciences for Life, Hiroshima University, Higashi-Hiroshima, Hiroshima, Japan; b Laboratory of BioDX, Genome Editing Innovation Center, Hiroshima University, Higashi-Hiroshima, Hiroshima, Japan; University of California, Riverside

## Abstract

We report the genome sequence of Ulva prolifera, which originated from the Yoshinogawa River in Japan, using Oxford Nanopore Technologies MinION and Illumina sequencing reads. The genome assembly size is 103.8 Mbp, consisting of 142 scaffolds with an *N*_50_ value of 4.11 Mbp.

## ANNOUNCEMENT

Ulva prolifera (Sujiaonori in Japanese) is one of the most important edible green alga in Japan. It grows in brackish river mouths; however, natural populations of U. prolifera in Japan have been decreasing, and a tank cultivation system has been developed for sustainable production (1). U. prolifera is also one of the sources of troublesome green tide (2). The genome sequence of U. prolifera collected from the Yellow Sea was recently reported (3); however, it is known that brackish strains and bloom-forming marine habitat strains show different characteristics, and new subspecies for the latter strains have been proposed (4, 5).

An U. prolifera sample for genome sequencing was collected from a land culture at Hashirijima Island (Hiroshima, Japan) that was originally derived from the Yoshinogawa River (Tokushima, Japan). The thallus, with a length of >20 cm, was cut into small pieces, frozen in liquid nitrogen, and finely powdered using a Micro Smash MS-100 cell disruptor (Tomy Seiko). Genomic DNA (gDNA) was extracted using the NucleoBond high-molecular-weight (HMW) DNA kit (Macherey-Nagel), and we did not perform either DNA shearing or size selection. A Nanopore sequencing library was prepared from 1 μg of gDNA using a ligation sequencing kit (SQK-LSK110; Oxford Nanopore Technologies [ONT]). Nanopore sequencing was performed on an ONT MinION sequencer using a R9.4.1 flow cell (FLO-MIN106D). One-half volume of the library was sequenced for 24 h, and the other one-half volume of the library was sequenced for 22 h after a flow cell wash step. The raw FAST5 files obtained for the two sequencing runs were base called using Guppy v6.0.1 (ONT) with a high-accuracy model (Table 1). An Illumina sequencing library was prepared using the NEBNext Ultra DNA library preparation kit for Illumina (New England Biolabs) and sequenced on an Illumina NovaSeq 6000 system in the 150-bp paired-end mode (Table 1).

**TABLE 1:** Summary of the sequence read and genome assembly data

Parameter	Finding
Nanopore sequencing	
No. of reads	5,280,010
No. of bases	17,282,510,862
Avg read length (bp)	3,273.2
Read *N*_50_ (bp)	9,388
Illumina sequencing	
Total no. of reads	74,904,442
Total no. of bases	11,235,666,300
Genome assembly	
Total length (bp)	103,843,212
No. of scaffolds	142
Scaffold *N*_50_ (bp)	4,106,524
Scaffold *L*_50_	8
Largest scaffold (bp)	9,543,602
No. of contigs	143
Contig *N*_50_ (bp)	4,106,524
Contig *L*_50_	9
Largest contig (bp)	9,355,488
GC content (%)	55.57

The base-called Nanopore reads were trimmed by fastp v0.23.2 (6) to remove low-quality head reads, and reads longer than 1,000 bp were collected (15,851 Mbp after trimming) for subsequent analysis. The Nanopore reads were assembled using NECAT v0.0.1_update20200803 (7) following a modified method (8). First the draft assembly by NECAT was polished with Medaka v1.6.0 (https://github.com/nanoporetech/medaka) using Nanopore reads, and then redundant haplotypes were removed with Purge Haplotigs v1.1.2 (9) using the Nanopore reads mapped with minimap2 v2.23 (10). Finally, a NECAT bridging step was performed using the primary contigs generated by Purge Haplotigs as a replacement for the draft assembly by NECAT. The bridged contigs from NECAT were polished three times with HyPo v1.0.3 (11) using the Illumina reads (after trimming with fastp v0.23.2) mapped with BWA-MEM v0.7.17 (12). Contigs that showed >90% identity and coverage against mitochondrial or chloroplast RefSeq sequences of *Ulva* species by BLASTn v2.12.0 were removed as organellar contigs. BESST v2.2.8 (13, 14) was used for scaffolding of the contigs using the Illumina reads mapped with BWA-MEM v0.7.17, and the final assembly was obtained (Table 1). Only two contigs were scaffolded with a 1-bp gap by BESST. The largest sequence (9,543,602 bp) (Table 1) was generated by this step.

Genome completeness analysis with BUSCO v5.2.2 (15) using the chlorophyta_odb10 data set (5 August 2020) and default settings showed 80.5% completeness (71.8% single-copy and 8.7% duplicated). Our BUSCO analysis of previously published U. prolifera (3) and Ulva mutabilis (16) genomes with the same settings showed relatively lower completeness scores (77.2% and 79.2%, respectively). Future studies will annotate genes to analyze this genome in more detail.

### Data availability.

This whole-genome shotgun project has been deposited in DDBJ under the accession numbers BRCE01000001 to BRCE01000142. The raw Nanopore and Illumina reads have been deposited in DDBJ under the accession numbers DRR361634 and DRR361635, respectively. Additional information on the parameter settings and draft assembly results with tested assemblers can be found at figshare (https://doi.org/10.6084/m9.figshare.19728862).
